# Lipids and Fatty Acids in Italian Durum Wheat (*Triticum durum* Desf.) Cultivars

**DOI:** 10.3390/foods8060223

**Published:** 2019-06-21

**Authors:** Valentina Narducci, Enrico Finotti, Vincenzo Galli, Marina Carcea

**Affiliations:** Research Centre for Food and Nutrition, Council for Agricultural Research and Economics (CREA), Via Ardeatina 546, 00178 Rome, Italy; valentina.narducci@crea.gov.it (V.N.); enrico.finotti@crea.gov.it (E.F.); vincenzo.galli@crea.gov.it (V.G.)

**Keywords:** durum wheat, fatty acids, grain, kernel, lipids

## Abstract

The level of variation in lipids and their fatty acids was determined in the grains of 10 popular durum wheat cultivars commercially grown in Central and Southern Italy. Samples were harvested for two consecutive years to account for differences due to changes in climatic conditions. Total fat content was determined by means of the International Association of Cereal Science and Technology (ICC) Standard Method No. 136, whereas the fatty acid profile was determined by gas chromatography. Total lipid content ranged from 2.97% to 3.54% dry basis (d.b.) in the year 2010 and from 3.10% to 3.50% d.b. in the year 2011, and the average value was 3.22% d.b. considering both years together. Six main fatty acids were detected in all samples in order of decreasing amounts: linoleic (C18:2) > palmitic (C16:0) ≈ oleic (C18:1) > linolenic (C18:3) > stearic (C18:0) > palmitoleic (C16:1). Significant variations in the levels of single acids between two years were observed for three samples. These results will be very useful in the updating of food composition databases in general and will help authorities to set proper quality standards for wholegrain flours and products where the germ should be preserved, considering also the recent interest of industry and consumers for these kinds of products.

## 1. Introduction

Durum wheat (*Triticum durum* Desf.) kernels contain about 2.4–3.8% dry basis (d.b.) of lipids [1]. Roughly two thirds (66%) of them are contained in the germ, 15% are in the bran (particularly in the aleuronic layer), and about 20% are distributed in the endosperm, partly within the starch granules. From a chemical point of view, the most abundant fraction is composed by nonpolar lipids, which are mainly storage acylglycerols. Phospholipids, glycolipids and other classes are present in lesser amounts. The fatty acids of wheat lipids are mostly unsaturated (C18:2, C18:1, C18:3 and C16:1) and two of them are essential (linoleic and linolenic). This increases the value of wheat lipids for human nutrition, because essential fatty acids are precursors of important classes of biomolecules in the human body (like prostaglandins and membrane phospholipids) and are involved in metabolic processes like regulation of blood lipid levels, particularly cholesterol [1,2,3].

Lipid content, lipid classes and fatty acid levels in wheat kernels depend on a set of factors, some of which are genetic, such as species and variety [4], whereas others depend on the environment and are related to pedoclimatic conditions, agronomic practices and maturity level [1,4,5]. For example, durum wheat and hard red wheat generally have a higher lipid content than soft white wheat and the levels of fatty acids are different in durum and in soft wheat. In regard to climatic conditions, it has been seen that cold weather favors an increase of lipid content in wheat and a higher degree of unsaturation in fatty acids due to the need for membrane fluidification [6]. Other kinds of biotic and abiotic stresses can influence the level of saturated and unsaturated fatty acids in plants [7]. Moreover, different extraction and analytical methods can also account for the differences found in the literature [1,8]. Notwithstanding the number of samples analyzed, we can assume that data about fatty acid levels in durum wheat are abundant in the literature, but it is difficult to have a clear idea of their content and to make comparisons for a number of reasons: (i) different authors report fatty acids as percentage, alternatively referring to: (1) total lipids, (2) total fatty acids, or (3) kernel weight (in addition, some authors analyze germ oil and others analyze whole kernels); (ii) authors interested in statistic elaborations (e.g., in order to investigate variation factors or to look for discriminating parameters) often report charts and graphs rather than tables of data; (iii) cultivars are different in different countries and new ones are constantly bred; and (iv) databases do not always report the sample numerosity and the standard variation of the means.

In this work, the content and level of variation in lipids and of their fatty acids in the durum wheat kernels commercially grown in Italy (where durum wheat is an important cereal crop mainly used for pasta manufacturing) were assessed. For this reason, we selected 10 cultivars amongst the most commonly grown for pasta making. Samples were collected in several locations of Central and Southern Italy to account, at least partially, for differences due to different pedoclimatic environments; Southern Italy is characterized by milder winters and warmer springs and summers with respect to Central Italy, however both areas are considered highly suitable for durum wheat cultivation. Moreover, crops from two consecutive years were collected from the same fields.

The knowledge generated by this research will be very useful in the updating of food composition databases in general and will help authorities in setting proper quality standards for wholegrain flours and products where the germ should be preserved, considering also the recent interest of industry and consumers for these kinds of products and the lack, in several cases, of specific legislation.

## 2. Materials and Methods

### 2.1. Samples and Sample Preparation

Representative samples of durum wheat grains, belonging to 10 cultivars selected amongst the most frequently grown in Italy, were collected at harvest for two consecutive years (2010–2011) in 10 different locations of Central and Southern Italy (Table 1). Eight samples came from the Central regions of Italy (Tuscany and Marche) whereas twelve were from different locations in the Sicilian region, in the South. All locations belong to the area traditionally dedicated to durum wheat cultivation in Italy.

Durum wheat in Italy is grown under rain-fed production: it is planted in late autumn or early winter and harvested in early summer, which often leads to limited rainfall and high temperatures, resulting in water stress during grain filling. Crop rotation and balanced nutrient management (mainly nitrogen and phosphorus, pre-sowing and topdressing fertilization) are practiced to ensure that the crop produces the greatest possible high-quality yield with the moisture that is available. The main climate factors influencing durum wheat crop quality are rainfall and temperature during the growing season. Data on these two factors of the years 2009–2011 in Central and Southern Italy can be found in the reports by the Italian High Institute for Environmental Protection and Research (ISPRA,) [9,10,11].

Fifty grams of each cleaned sample were milled by means of a Cyclotec laboratory mill (Foss-Tecator, Hillerød, Denmark) equipped with a 0.5 mm screen, to obtain wholemeal flours that were used for the subsequent analyses.

### 2.2. Chemicals

Chloroform, ethyl alcohol (96% *w/w*), methanol, *n*-hexane, formic acid (99% *w/w*) hydrochloric acid (37% *w/w*) and anhydrous sodium sulphate were of analytical grade and were purchased from Carlo Erba (Milan, Italy). Boron trifluoride (approximately 10% *w/w* in methanol for gas chromatography (GC) derivatization) was purchased from Sigma-Aldrich (St. Louis, MO, USA). Fatty acid standards (C16:0, C16:1, C17:0, C18:0, C18:1, C18:2, C18:3) were also purchased from Sigma-Aldrich.

### 2.3. Analyses

Moisture of wholemeal flours was determined by oven drying at 130 °C according to the ICC Standard No. 110/1 [12].

Total fat was determined by hydrolysis in formic acid and hydrochloric acid at 75 °C reflux for 20 min followed by extraction in hexane and evaporation, according to the ICC Standard No. 136 [12].

The fatty acid profile was determined by gas chromatography (GC). About 5 g of wholemeal flour (in duplicate) was introduced in a Corning tube and suspended in 10 mL of chloroform–methanol 2:1 acidulated with 6 N HCl. A magnetic bar was added, and the tube was left to extract overnight at room temperature on a magnetic stirrer. The mixture was filtered through Whatman Grade 1 (1–11 μm) filter paper into an oven dried flask, then the solvent was evaporated by nitrogen flux followed by oven drying at 30 °C. The contents of the flask were re-dissolved in chloroform–methanol 2:1 to a volume of exactly 10 mL, then an aliquot was derivatized according to Zweig and Sherma [13] as follows: 100 μL of this solution was introduced into a Corning tube containing 3 mL of methanol and a few boiling stones, then 0.5 mL of BF_3_–methanol (10% *w/w*) was added and the tube caps were loosely screwed. The tubes were put onto a heating plate in a water bath and left to gently reflux at 72 °C for 30 min. Following this, the reaction was quenched with 2 mL of water, then the mixture was cooled to room temperature and extracted three times with 3 mL of *n*-hexane. The hexane extracts were reunited into a vial and finally the hexane was evaporated by nitrogen flux. The vial was stored under nitrogen at −18 °C for a few days. Immediately prior to GC analysis, the contents of the vial were re-dissolved in 300 μL of hexane and 2 μL were injected. The GC instrument was an HP 5890 equipped with a Supelco (Sigma-Aldrich, St. Louis, MO, USA) SPB^®^-PUFA (poly unsaturated fatty acids) column of 30 m length and a flame ionization detector (F.I.D.). The instrumental analysis was run according to Finotti et al. [14]: 50 °C for 1 min, ramp of 10 °C/min until 160 °C, stay at 160 °C for 1 min, ramp of 2 °C/min until 240 °C. The detected peaks were individuated by comparison with chromatograms of standards (C16:0, C16:1, C18:0, C18:1, C18:2, C18:3) and quantified by using C17:0 as an internal standard.

### 2.4. Statistics

The Shapiro–Wilks normality test, *F*-test for homogeneity of variance, Student’s *t*-test and Friedman test followed by Wilcoxon pairwise comparisons were performed by means of the PAleontological STatistics (PAST) statistical package [15]. Two-way ANOVA followed by Tukey’s test (only in cases with a normal variable and homogeneous variances) and box-plots were performed by means of StatSoft Statistica 8.0 (TIBCO Software, Palo Alto, CA, USA). Calculations were performed by means of Microsoft Excel (Redmond, Washington State, USA).

## 3. Results

### 3.1. Total Lipids

Total lipids ranged from 2.97% to 3.54% d.b. in the year 2010 and from 3.10% to 3.50% d.b. in the year 2011, and the average value was 3.22% d.b. considering both years together (Table 2). The moisture content of grains ranged between 10.5% and 12.3% and the average was 11.4% (Table 2). Total lipid content was strongly dependent on the combination of cultivar (cv)/growing site (*p* < 0.01) and to a minor extent on the growing year (*p* < 0.05), whereas the interaction cv/site × year was not a statistically significant factor of variation. In any case, differences were very small: up to 0.57 between samples of different cultivars and up to 0.18 between years for samples of a same cv/site (Table 2). Differences between years for samples of the same cv/site were not significant. The total lipid values found in this study are in line with those reported by the USDA National Nutrient Database (2.8 g/100 g d.b. for product N. 20076 “wheat, durum”, mean of 18 samples, standard error 0.060) and by the Italian food composition tables (3.3 g/100 g d.b. for “durum wheat”) compiled by the Italian National Institute for Research on Food and Nutrition (INRAN) [16,17]. If we take into account the geographical separation into Central and Southern Italy, we can say that the average total lipid values for all samples were 3.24% and 3.21% d.b. respectively, whereas the range of values was 2.97–3.54% for Central Italy and 3.09–3.41% d.b. for Southern Italy.

### 3.2. Fatty Acid Profile

**Figure 1:**
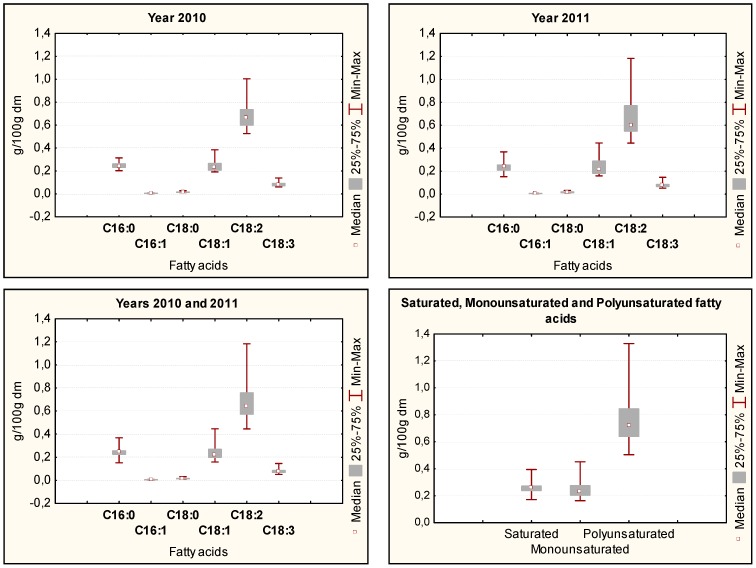
Box plot (percentiles) of fatty acids in samples of Italian durum wheat (10 cultivars, grown in the same location for two consecutive years).

Six main fatty acids were detected in all samples, as expected. In order of decreasing amounts, they are: linoleic (C18:2) > palmitic (C16:0) ≈ oleic (C18:1) > linolenic (C18:3) > stearic (C18:0) > palmitoleic (C16:1). This can be clearly seen from the box plot elaboration reported for each separate year and for the two years together (Figure 1). This distribution did not change whether considering both years separately or together. Detailed data of fatty acids in all samples are reported in Table 3.

Linoleic acid (C18:2) was present in amounts ranging from 0.50–1.14 g/100 g d.b. throughout all samples, with a mean of 0.68 and a standard deviation (SD) of 0.16 (Table 3). For comparison, the USDA National Nutrient Database reports 1.04 g/100 g d.b. for product N. 20076 “wheat, durum” and the INRAN food composition tables report 1.36 g/100 g d.b. for durum wheat. Neither database reports any information on standard errors for all acids.

Palmitic (C16:0) and oleic (C18:1) acids were detected in equal amounts. Palmitic acid ranged from 0.17–0.36 g/100 g d.b., mean 0.24 (SD 0.04) and oleic acid ranged from 0.17–0.43 g/100 g d.b., mean 0.24 (SD 0.07). The USDA reports 0.51 g/100 g d.b. for palmitic acid and 0.40 g/100 g d.b. for oleic acid, whereas the INRAN database reports 0.47 g/100 g d.b. and 0.38 g/100 g d.b., respectively (Table 3).

Linolenic acid (C18:3) ranged from 0.06–0.14 g/100 g d.b., mean 0.08 (SD 0.02). The USDA and the INRAN databases report 0.05 g/100 g d.b. and 0.11 g/100 g d.b., respectively. Stearic acid (C18:0) ranged from 0.01–0.03 g/100 g d.b., mean 0.02 (SD 0.005). The USDA and the INRAN databases report, for this acid, 0.03 g/100 g d.b. and 0.02 g/100g d.b. respectively. Finally, palmitoleic acid (C16:1) was detected in very small amounts, ranging from 0.004–0.007 g/100 g d.b., mean 0.005 (SD 0.001). Both the USDA and INRAN databases report 0.01 g/100 g d.m. for this acid.

A series of *t*-tests, performed for each fatty acid on each pair of samples from the same cv/site between the two growing years, showed a significant difference between the years 2010 and 2011 in a few cases only, namely: all acids except C16:1 varied in Ancomarzio SI and Iride AG; only the acids C18:1, C18:2 and C18:3 varied in Ciccio EN (Table 3).

### 3.3. Saturated and Unsaturated Fatty Acids

As expected, polyunsaturated fatty acids were preponderant over saturated and monounsaturated fatty acids in all samples (*p* < 0.01, Friedman test; see Figure 1), ranging from 0.57–1.28 g/100 g d.b. (Table 3). Total monounsaturated and total saturated, whose levels were roughly similar (*p* < 0.01), covered from 0.18–0.44 g/100 g d.b. and from 0.19–0.39 g/100 g d.b., respectively. The unsaturated/saturated ratio ranged from 3.15–4.44 g/100 g d.b. considering all samples, with a mean of 3.83 (SD 0.38). This mean is higher than that reported by USDA (3.0) and INRAN (3.5). A series of *t*-tests, performed on each pair of samples from the same cv/site grown in different years, showed a significant difference for the unsaturated/saturated ratio between years in only three cases (Ancomarzio SI, Iride AG and K26 EN) (Table 3).

## 4. Discussion

Total lipids were in line with the values reported by the USDA and the INRAN databases (nearer to the Italian value) and it was not possible to detect any difference between the geographical areas of Central and Northern Italy.

In regard to fatty acid composition, even if Bottari et al. in 1999 [18] observed the presence of more than 60 peaks by gas chromatography and mass spectrometry (GC-MS) and identified fatty acids with even numbers of carbon atoms from C12 to C30 as well as C15 and C17, the major fat components were saturated and unsaturated C16 and C18 and particularly C16:0, C18:1 and C18:2, which together represented around 90% of the total. 

Actually, the USDA database (but not the INRAN one) and other works also report small amounts of C14:0 in durum wheat kernels (USDA 0.003 g/100 g fresh matter, corresponding to 0.0035 g/100 g d.b.). We did not detect this acid, as it was at the limit of detection of our method. There are publications reporting other fatty acids as well (i.e., C17, C20, C22 and C24), some in kernels (Beleggia et al. [5] who uses a GC-MS instrument) and others in germ oil [19,20]. However, only C16:0, C18:0, C18:1, C18:2 and C18:3 are constantly reported by all published works and are regarded as the most important ones in durum wheat, with others amounting to about 1–2% in total [1].

For all fatty acids except C18:3 and for total saturated, total monounsaturated and total polyunsaturated acids, the mean calculated for our samples was lower than the values reported by USDA and INRAN (roughly two thirds–half, *p* < 0.01 against a hypothetical value; see Table 3). However, the range of the detected values contained the reference values, except for C16:0 and for total saturated acids, for which the detected range extended entirely below the USDA and INRAN means. Neither database reports the standard deviations for fatty acids in durum wheat and only the USDA one reports sample numerosity (that is, 29); in this latter case, a certain width around the reported value can be supposed, but it is not quantified. On the contrary, for the unsaturated/saturated ratio, the range of the detected values extends entirely above the mean reported by USDA and contains that reported by INRAN. As a matter of fact, there are notable differences between the two references used. The INRAN values are equal to or higher than the USDA values for the considered variables, in particular for C18:0 (+50%), C18:2 (+31%), C18:3 (+120%), total polyunsaturated acids (+34%) and unsaturated/saturated ratio (+17%).

All the reported differences can be explained by the differences in genetic characteristics, pedoclimatic conditions, agronomical treatments and analytical procedure, as stated in the Introduction. In particular, Beleggia et al. [5] identified the interaction genotype × year × treatment as the main contributor to the variability of the fatty acid levels observed in 24 durum wheat samples, especially for linoleic, oleic and stearic acids. Armanino et al. [4] linked the fatty acid profile of 135 samples of durum wheat to the cultivar, the geographic origin and the harvest year. The variation in saturated and unsaturated fatty acids within the same variety is also associated with various kinds of biotic and abiotic stresses, like low or high temperature, salt, drought, pathogens and others [6,7].

Also, in our study, different conditions related to location and climatic factors can account for some of the observed variability in lipid parameters. In fact, from the ISPRA reports [9,10,11], we can briefly say that in both areas of Italy (Central and South), temperatures were similar in the first part of the two growing seasons (October–December 2009 and 2010), except the month of December which was warmer in 2009 than in 2010. In the second part of the growing season (January–June, particularly April–June), the Central area showed warmer temperatures in 2011 than in 2010. In regard to precipitation, the first growing season (2009) started with a lesser amount of rain in October–December with respect to the second one (2010) and continued with a higher amount of rain in the January–June period. This happened both in Central and Southern Italy.

## 5. Conclusions

This work contributes to the knowledge on the content and variability of total fats and of the main fatty acids in durum wheat kernels. The values obtained in this study are also compared with reference values from national and international databases. In this paper, the use of standard methods of analysis, statistical data (numerosity of samples, mean, standard errors) and the specification of all the elements that allow for conversion of results into different units of measure (g/100 g dry or wet sample, g/100 g fat matter) make this data very useful in the compilation of databases and easy to compare with other data. Moreover, updated data on lipids are needed to set proper quality standards for products such as wheat wholegrain flours and foods where the presence of germ is desirable.

## Figures and Tables

**Table 1:** Durum wheat sample specifications: cultivar, region and location.

Cultivar	Region	Location
Ancomarzio	Tuscany (Central Italy)	Siena (SI)
Creso	Tuscany (Central Italy)	Pisa (PI)
Dylan	Marche (Central Italy)	Macerata (MC)
Rusticano	Marche (Central Italy)	Ancona (AN)
Bronte	Sicily (Southern Italy)	Palermo (PA)
Ciccio	Sicily (Southern Italy)	Enna (EN)
Duilio	Sicily (Southern Italy)	Trapani (TP)
Iride	Sicily (Southern Italy)	Agrigento (AG)
K26	Sicily (Southern Italy)	Enna (EN)
Simeto	Sicily (Southern Italy)	Catania (CT)

**Table 2:** Moisture and total lipids in the grains of 10 Italian durum wheat cultivars grown in different locations for two consecutive years.

Cultivar and Location		Moisture (g/100 g)		Total Lipids (g/100g d.b.)			
		2010	2011	2010	2011	Difference 2011–2010	
Central Italy	Ancomarzio SI	11.2	11.0	3.11 ^ef^	3.25 ^cde^	0.14	ns
	Creso PI	11.8	11.4	3.24 ^cde^	3.25 ^cde^	0.01	ns
	Dylan MC	11.7	11.7	3.54 ^a^	3.50 ^ab^	−0.04	ns
	Rusticano AN	12.3	12.0	2.97 ^f^	3.10 ^ef^	0.13	ns
Southern Italy	Bronte PA	11.3	11.1	3.09 ^ef^	3.28 ^bcde^	0.18	ns
	Ciccio EN	11.5	10.8	3.39 ^abcd^	3.41 ^abc^	0.02	ns
	Duilio TP	11.2	11.4	3.11 ^ef^	3.15 ^def^	0.05	ns
	Iride AG	11.0	10.5	3.31 ^abcde^	3.24 ^cde^	−0.07	ns
	K26 EN	11.3	11.0	3.10 ^ef^	3.24 ^cde^	0.14	ns
	Simeto CT	11.7	11.6	3.13 ^ef^	3.10 ^ef^	−0.02	ns

^abcdef^: different letters correspond to significant differences (*p* < 0.05) according to 2-way ANOVA and Tukey’s test. ns: not significant.

**Table 3:** Fatty acids in 10 Italian durum wheat cultivars, grown in different locations for two consecutive years (g/100 g sample, d.m.).

Sample	Total Lipids	C16:0	C16:1	C18:0	C18:1	C18:2	C18:3	Saturated	Monoun- Saturated	Polyun- Saturated	Ratio Unsaturated/ Saturated
Ancomarzio (SI) 2010	3.01	0.28	0.006	0.02	0.28	0.80	0.10	0.30	0.29	0.90	3.96
Ancomarzio (SI) 2011	3.03	0.23	0.004	0.01	0.18	0.54	0.06	0.24	0.18	0.60	3.24
		*			*	*	*				*
Creso (PI) 2010	3.02	0.23	0.004	0.02	0.24	0.68	0.07	0.25	0.24	0.75	3.97
Creso (PI) 2011	3.02	0.17	0.004	0.02	0.20	0.51	0.06	0.19	0.21	0.57	4.05
											
Dylan (MC) 2010	3.05	0.26	0.007	0.01	0.28	0.68	0.07	0.27	0.28	0.75	3.80
Dylan (MC) 2011	3.05	0.27	0.005	0.02	0.34	0.84	0.08	0.29	0.34	0.92	4.39
											
Rusticano (AN) 2010	3.00	0.23	0.005	0.02	0.22	0.65	0.08	0.25	0.23	0.72	3.87
Rusticano (AN) 2011	3.01	0.26	0.006	0.02	0.30	0.85	0.09	0.28	0.30	0.94	4.42
											
Bronte (PA) 2010	3.01	0.30	0.006	0.02	0.33	0.89	0.13	0.32	0.34	1.02	4.20
Bronte (PA) 2011	3.03	0.22	0.004	0.01	0.18	0.50	0.07	0.23	0.18	0.57	3.24
											
Ciccio (EN) 2010	3.04	0.24	0.004	0.02	0.25	0.73	0.09	0.26	0.25	0.83	4.11
Ciccio (EN) 2011	3.04	0.20	0.004	0.01	0.17	0.54	0.07	0.21	0.18	0.61	3.69
					*	**	**				
Duilio (TP) 2010	3.01	0.22	0.004	0.01	0.20	0.60	0.08	0.23	0.20	0.68	3.87
Duilio (TP) 2011	3.02	0.23	0.004	0.01	0.22	0.64	0.08	0.24	0.22	0.73	3.94
											
Iride (AG) 2010	3.03	0.23	0.007	0.01	0.22	0.59	0.07	0.24	0.22	0.66	3.62
Iride (AG) 2011	3.02	0.36	0.007	0.03	0.43	1.14	0.14	0.39	0.44	1.28	4.44
		**			**	**	**				*
K26 (EN) 2010	3.01	0.24	0.005	0.02	0.21	0.54	0.06	0.26	0.21	0.60	3.15
K26 (EN) 2011	3.02	0.24	0.005	0.01	0.22	0.59	0.07	0.26	0.23	0.66	3.48
							*				**
Simeto (CT) 2010	3.01	0.23	0.005	0.01	0.21	0.64	0.08	0.25	0.21	0.72	3.76
Simeto (CT) 2011	3.01	0.25	0.006	0.01	0.20	0.63	0.08	0.26	0.21	0.71	3.48
											
Max	3.00	0.36	0.007	0.03	0.43	1.14	0.14	0.39	0.44	1.28	4.44
Min	3.05	0.17	0.004	0.01	0.17	0.50	0.06	0.19	0.18	0.57	3.15
											
Mean	3.02	0.24	0.005	0.016	0.24	0.68	0.08	0.26	0.25	0.76	3.83
SD	0.014	0.04	0.001	0.005	0.07	0.16	0.02	0.04	0.07	0.18	0.38
											
Durum wheat USDA ^‡^	2.8	0.47	0.01	0.02	0.38	1.04	0.05	0.50	0.39	1.10	3.0
Durum wheat INRAN ^‡^	3.3	0.51	0.01	0.03	0.40	1.36	0.11	0.54	0.41	1.47	3.5

Asterisks indicate significant difference between the year 2010 and 2011, * *p* < 0.05, ** *p* < 0.01 (*t*-test). ‡: values on dry basis, calculated by the authors from original data in USDA and INRAN databases that are expressed on as-is basis.
